# The role of hepatic stellate cells in the regulation of T-cell function and the promotion of hepatocellular carcinoma

**DOI:** 10.3892/ijo.2012.1497

**Published:** 2012-05-24

**Authors:** WENXIU ZHAO, WEIXUE SU, PENGHAO KUANG, LEI ZHANG, JIANMING LIU, ZHENYU YIN, XIAOMIN WANG

**Affiliations:** Department of Hepatobiliary Surgery, Zhongshan Hospital, Xiamen University, Research Institute of Digestive Diseases, Xiamen University; Xiamen, Fujian 361004, P.R. China

**Keywords:** hepatic stellate cells, immunosuppression, hepatocellular carcinoma, T cells

## Abstract

Hepatic stellate cells (HSCs) have immunosuppressive abilities and may be responsible for the occurrence and development of hepatocellular carcinoma (HCC). However, the mechanisms through which HSCs affect T-cell-mediated immune responses remain unclear. The aim of this study was to elucidate these mechanisms. We examined the effect of HSCs on T-cell proliferation and apoptosis, regulatory T cells (Treg cells) and T-cell-mediated cytotoxicity using mixed leukocyte reactions (MLRs). Furthermore, we examined the cytokines present in the supernatant and the effect of this supernatant on the proliferation and migration of cancer cells. Finally, we examined the effect of HSCs on HCC cells *in vivo*. We found that activated HSCs induced T-cell hyporesponsiveness, accelerated activated T-cell apoptosis, increased the number of Treg cells and inhibited T-cell-mediated cytotoxicity. HSCs also enhanced the expression of some cytokines and promoted the proliferation and migration of cancer cells. Furthermore, activated HSCs were able to induce HCC proliferation and Treg cells expansion *in vivo*. Activated HSCs may induce T cell anergy, thereby facilitating the immunologic escape of HCC cells.

## Introduction

Hepatocellular carcinoma (HCC) is a common malignancy worldwide. Of the numerous therapies available, hepatic resection is currently the first choice, although it is often accompanied by a poor prognosis. Another therapeutic option, liver transplantation, is not widely used as there is a shortage of livers available, and many patients succumb to the disease while waiting for a transplant (1). Several experts have focused on biotherapy for decades; however, the mechanism underlying HCC development remains unclear, making it difficult to develop effective therapies.

Following injury, non-parenchymal cells, especially hepatic stellate cells (HSCs), are activated and change from a quiescent state to myofibroblast-like cells. Activated HSCs can secrete cytokines and produce collagen (2,3), and they can potentially contribute to the repair of liver damage. However, the excessive activation of HSCs can induce liver fibrosis (4–6), which can progress to hepatocirrhosis or even HCC. As a result of these studies, it has been suggested that there may be a relationship between HSCs and HCC.

Recent studies have confirmed that HSCs have immunomodulatory activities and can inhibit T-cell-mediated tissue destruction and, notably, can prolong the survival of allografts (7–11). Furthermore, activated HSCs can promote the occurrence and development of HCC (12,13). In our previous study, we found that activated HSCs induce an immunosuppressive microenvironment that promotes HCC development *in vivo*(14). However, how HSCs affect T cells has yet to be elucidated. Additionally, whether T cells can still attack cancer cells when their functions are inhibited by HSCs also remains unknown.

In this study, we investigated whether HSCs could block the cancer-fighting ability of T cells and therefore promote the development of HCC *in vitro*. We found that activated HSCs induced T cell hyporesponsiveness by promoting T-cell apoptosis and expanding regulatory T cells (Treg cells), thereby changing the balance of the different T-cell populations in mixed leukocyte reactions (MLRs). In addition, HSCs inhibited the cytotoxic T cell-mediated lysis of tumour cells. Moreover, activated HSCs altered the cytokines that were released during the MLRs, and this alteration could promote the development of HCC.

## Materials and methods

### Animals

C57BL/6 (B6; H2b) and BALB/c (H2d) mice were purchased from the National Rodent Laboratory Animal Resources, Shanghai branch. All animals were maintained under specific pathogen-free conditions in the Laboratory Animal Centre of Xiamen University Medical Department and provided with rodent chow and tap water. All mice were 8–12 weeks old when used, and all experiments were performed following the Laboratory Animal Centre care guidelines.

### Isolation, culture, and identification of HSCs

HSCs were isolated and cultured as previously described (14,15). Cell viability was determined using trypan blue exclusion, and the appearance of the HSCs was verified using light microscopy. The purity of the HSCs was examined using desmin immunofluorescence. HSCs were obtained after passage for 2–3 generations, and the cells were plated on glass slides, fixed at 70–80% confluence, and stained with antibodies specific for desmin (NeoMarker) or α-smooth muscle actin (α-SMA, Abcam). The stained HSCs were examined using confocal microscopy.

### Isolation and culture of dendritic cells (DCs)

Bone marrow cells were isolated from C57BL/6 mouse femurs and tibias. After lysing the red blood cells with lysis buffer (Beyotime), the remaining cells were cultured in 6-well plates (Corning) in RPMI-1640 medium (Hyclone) supplemented with 10% fetal bovine serum (FBS) (complete RPMI-1640 medium) in the presence of mouse recombinant granulocyte-macrophage colony-stimulating factor (GM-CSF, 10 ng/ml, Hangzhou LongGene) and interleukin-4 (IL-4,10 ng/ml, Peprotech), as previously described (16). The media was replaced every 2 days. Non-adherent cells were spontaneously released from the proliferating cell clusters, and these floating cells were harvested on Day 7.

### Preparation of T lymphocytes

Enriched T cells were prepared as previously described (17) with minor modifications. Spleens were isolated from BALB/c mice under sterile conditions and pushed through a 75-*μ*m steel mesh. After the red blood cells were lysed, the remaining cells were placed in a nylon wool column and incubated for 45 min at 37°C in 5% CO_2_ in air. The non-adherent cells were then collected for experiments. The purity of the T cells was >90%.

### T-cell proliferation assay

One-way MLRs assays were performed in triplicate in 96-well microculture plates (Corning). First, HSCs were treated with mitomycin C (40 *μ*g/ml) for 25 min and washed twice before use. Nylon wool-purified splenic T cells from BALB/c mice were used as responders (2×10^5^) and were co-cultured with mitomycin C (20 *μ*g/ml)-treated DCs at a ratio of 10:1. To evaluate the ability of HSCs to inhibit T-cell proliferation, mitomycin C-treated HSCs were added at the beginning of each MLRs at different ratios (T:HSCs = 10:1, 20:1, 40:1, or 80:1), while the control had no HSCs added. All of the cells were cultured in complete RPMI-1640 medium for 72 h before proliferation was measured using the BrdU Cell Proliferation Assay (Roche) and a microtitre plate reader at 450 nm. The supernatant was collected separately.

### Hepa1-6-cell proliferation assay

The supernatant (referred to as ‘conditioned medium’) from the MLRs was collected. To determine the effect of the conditioned medium on tumour cell proliferation, Hepa1-6 cells (purchased from the Shanghai Cell Bank, Chinese Academy of Sciences) were plated in triplicate at 4×10^3^ cells/well in 96-well plates and allowed to adhere overnight. The culture medium was then replaced with the conditioned medium. After 72 h, cell proliferation was measured using the BrdU Cell Proliferation Assay. The absorbance values are directly correlated with the amount of DNA synthesis and, thus, with the number of proliferating cells in culture.

### In vitro T-cell apoptosis assay

Purified T cells and mitomycin C-treated DCs with or without mitomycin C-treated HSCs were co-cultured T:DC:HSCs at a ratio of 20:2:1 in 24-well plates (Corning). After 72 h, the cells were collected, stained with fluorescein isothiocyanate (FITC)-conjugated Annexin V and propidium iodide (PI) using the Apoptosis Analysis Kit (Keygen, Nanjing), according to the manufacturer’s protocol, and analysed using flow cytometry. In addition, the supernatant was collected.

### Detection of Treg cells

The 3 types of cells (T cells, DCs, and HSC) were co-cultured as described for the apoptosis assay. After 72 h of incubation, the cells were collected and examined using the Mouse Regulatory T Cell Staining Kit (eBioscience) according to the manufacturer’s instructions. The supernatant was also collected.

### In vitro cytotoxic T-lymphocyte assay

Hepa1–6 cells were collected and subjected to 4 cycles of snap freeze-thaw to obtain tumour lysates that could be used to pulse DCs. DCs were stimulated using the tumour lysate on day 4 of culture, incubated for another 48 h, and collected as stimulators. As targets, Hepa1–6 cells were labelled with CFSE (10 *μ*M). The effectors, stimulators, and targets were plated at a ratio of 50:5:1 (T:DC:Hepa1–6, T cells = 2×10^6^). Mitomycin C-treated HSCs were added to the experimental groups at the start of the experiment. After co-culturing the cells for 72 h, the cells were harvested, stained using PI (1 mg/ml) in a total volume of 500 *μ*l, and examined using flow cytometry. The CFSE/PI double-positive cells were considered as dead Hepa1–6 cells; therefore, the percentage of CFSE/PI double-positive cells was as the percentage of the mortality rate of Hepa1–6.

### Transwell assay

Transwell migration chambers (Corning) were used to observe the migration of HCC cells. Hepa1–6 cells (5×10^4^) in 200 *μ*l serum-free medium were added to the upper chamber, and the supernatant (800 *μ*l) collected from the MLRs experiments with or without HSCs was added to the lower chamber. The cells were allowed to migrate for 18 h at 37°C in a 5% CO_2_ atmosphere. The non-migrating cells on the upper surface of the membrane were removed using a cotton swab. The remaining cells were fixed in methanol, stained using crystal violet, and air-dried. The number of migrating cells on each membrane was counted using a microscope.

### Cytokine analysis

To measure cytokine production in the MLRs, the collected supernatant was lyophilised to a total volume of 1 ml and analysed using the Mouse Cytokine Array Panel A (R&D), as recommended by the manufacturer. The data were analysed using image analysis software and the expression of cytokines with fold change >±1.5 in the experimental group are shown.

### Animal model

*In vivo* tumour growth was measured using a previously described animal model (18). Briefly, C57BL/6 mice were subcutaneously injected in their backs with a 0.1 ml cell suspension containing either 1×10^6^ Hepa1–6 cells or a mixture of 1×10^6^ Hepa1–6 cells and 4×10^5^ activated HSCs. Each experimental group consisted of 6 animals. The tumour growth kinetics were monitored by measuring the length and width of the tumour mass at the inoculation site. At the end of the experiment, the mice were euthanised, and the tumours were collected and stored for subsequent analysis.

### Histochemistry and immunohistochemistry

Paraffin-embedded tissue samples were serially sectioned and immunohistochemically examined using antibodies against PCNA (Cell Signalling) or stained using an FITC-conjugated anti-Foxp3 antibody. Slides were visualised and photographed using a Leica DM2500 light and fluorescence microscope.

### Statistical analysis

The data were analysed using SPSS software (version 13.0). The results are expressed as the mean ± SEM. Statistical analyses were performed using a one-way ANOVA and a Student’s t-test. The statistical significance level was set at 0.05.

## Results

### Culture and identification of HSCs

**Figure 1:**
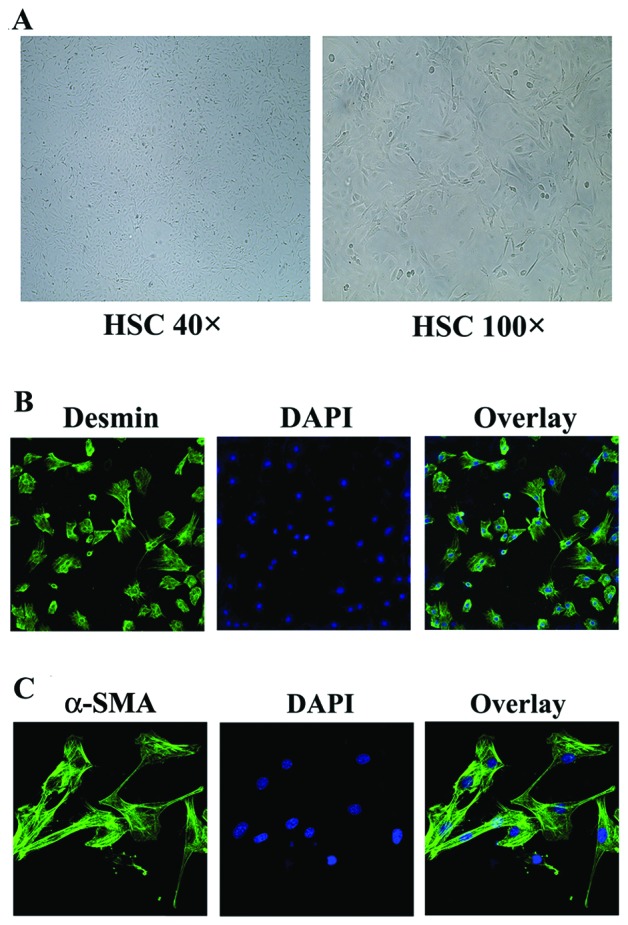
Morphology and identification of HSCs. (A) HSCs were isolated from C57BL/6 mouse livers. After 2 weeks of *in vitro* culture, the HSCs gradually exhibited a myofibroblast-like shape. (B and C) HSCs were collected and stained using antibodies specific for desmin (B) or α-SMA (C), followed by an FITC-conjugated secondary antibody; the nucleus was stained using DAPI. The purity of the HSCs was based on desmin staining, and 500 cells were scored. The expression of α-SMA was used as an indicator of activation. Magnification: B, ×200; C, ×400.

Previous studies have demonstrated that desmin, the gold standard for identifying HSCs, is expressed in both quiescent and activated HSCs (18); however, α-SMA has only been detected in activated HSCs (6). Following isolation and culture, the HSCs gradually displayed a myofibroblast-like shape (Fig. 1A) and became mature. The purity of the HSCs was >90% based on desmin staining (Fig. 1B). After *in vitro* culture for 14 days, the HSCs were activated and strongly expressed α-SMA (Fig. 1C).

### HSCs inhibit T-cell responses

**Figure 2:**
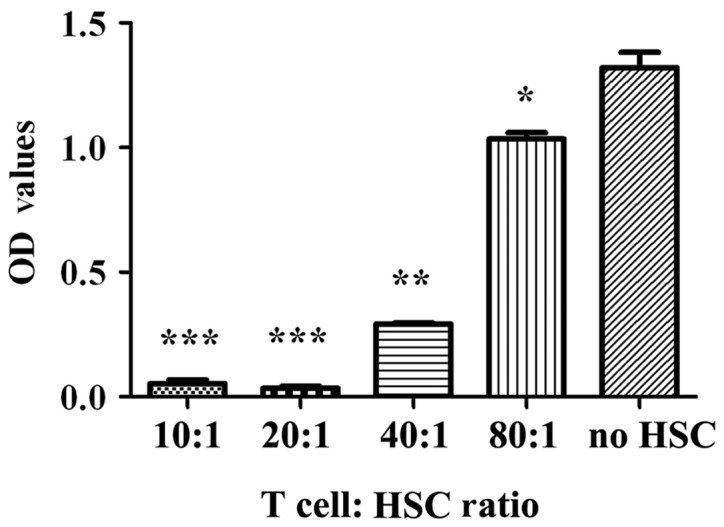
Activated HSCs inhibit T-cell proliferation. In each group, 2×10^5^ nylon wool-purified T cells were cultured with DCs with or without HSCs. The ratio of T cells:DCs (10:1) was the same in all reactions, but the ratio between the T cells and HSCs varied. HSCs reduced the proliferation of T cells in a dose-dependent manner. There was no difference between the 10:1 and the 20:1 group. The data are expressed as the means ± SEM. ^*^p<0.05, ^**^p<0.01, and ^***^p<0.001.

To examine the effect of HSCs on the proliferation of T cells, we used one-way MLRs. As shown in Fig. 2, the inhibition of T-cell proliferation could be correlated with the T:HSC cell ratio in the culture. The highest level of HSC-mediated inhibition of T-cell proliferation was observed at a ratio of 20:1 (p<0.001). However, this inhibition did not increase when more HSCs were added to the culture (compare the proliferation in the 10:1 and 20:1 cultures, p>0.05).

### HSCs enhance T-cell apoptosis

**Figure 3:**
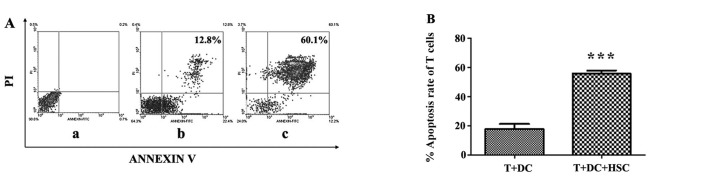
HSC-induced inhibition of T-cell responses is associated with T-cell apoptosis. Mitomycin C-treated activated B6 (H2b) HSC (40 *μ*g/ml mitomycin C) were added at the beginning of one-way MLRs containing nylon wool-purified splenic T cells (1×10^6^) from BALB/c (H2d) mice cultured with mitomycin C-treated B6 (H2b) DCs (20 *μ*g/ml mitomycin C) at a final T-cell:DC:HSC ratio of 20:2:1 for 3 days. (A) The apoptosis of activated T cells in the group with HSCs (panel c) was markedly higher than in the group without HSCs (panel b). However, the same trend was not seen in the Annexin V^+^ PI^−^ cells. Panel a, blank control; Panel b, T+DC; Panel c, T+DC+HSC. (B) Data are expressed as the mean ± SEM (^***^p<0.001).

We speculated that the HSC-induced T-cell hyporesponsiveness may result from the apoptosis of activated T cells. To determine the effect of HSCs on T-cell apoptosis, we seeded T cells, DCs, and mitomycin C-treated HSCs in a 24-well plate, and after 3 days of culture, the cells were stained using Annexin V and PI. As shown in Fig. 3A, the proportion of cells that were double-positive for Annexin V and PI staining increased significantly from 12.8 to 60.1% (Fig. 3B, p<0.001). These results confirm that HSCs greatly enhance T-cell apoptosis.

### HSCs promote the expansion of Treg cells

**Figure 4:**
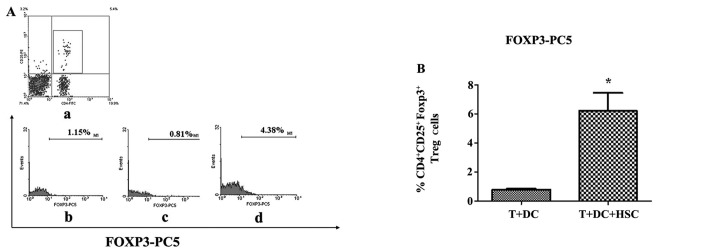
Activated HSCs induce the expansion of Treg cells. Cells were co-cultured in the same manner as the T-cell apoptosis assay. (A) HSCs increased the amount of CD4^+^CD25^+^FoxP3^+^ cells, and the percentage of Treg cells increased from 0.81 to 4.38%. Panel a, gated on CD4^+^CD25^+^ cells; Panel b, Isotype control staining for FoxP3; Panel c, T+DC; Panel d, T+DC+HSC. (B) The data are expressed as the mean ± SEM (^*^p<0.05).

Treg cells suppress T-cell responses *in vitro* via cell-cell contact and infectious tolerance. *In vivo*, Treg cells can also create a regulatory milieu via the secretion of IL-10 and/or TGF-β, which results in both antigen-specific and bystander immunosuppression (19). To determine whether the HSC-mediated immunosuppression occurred through Treg cells, we examined Treg cells using MLRs. As shown in Fig. 4A, the percentage of CD4^+^CD25^+^ FoxP3^+^ cells in the experimental group was higher than in the control group (4.38 vs. 0.81%, p<0.05). This result confirms that HSCs can expand the Treg cell population.

### HSCs inhibit the cytotoxic T lymphocyte response

**Figure 5:**
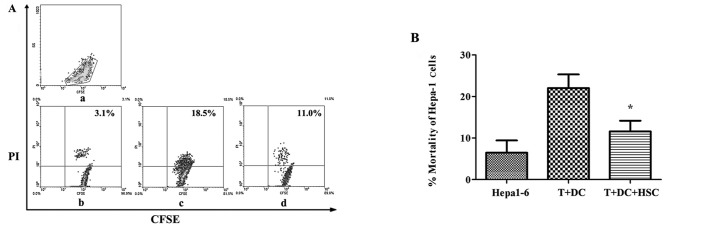
Activated HSCs inhibit the cytotoxicity of activated T cells against target cells. The cells were treated and cultured as described in Materials and methods. (A) HSCs inhibited the cytotoxicity of T cells. Activated T cells showed strong cytotoxicity against Hepa1–6 cells; however, the mortality rate of targets decreased when HSCs were added at the start of the MLRs. Panel a, detection of labelled Hepa1–6 cells; Panel b, the natural mortality of the target cells (Hepa1–6); Panel c, T+DC, the mortality of targets in the group without HSCs; Panel d, T+DC+HSC, the mortality of targets in the group with HSCs. (B) Data are expressed as the mean ± SEM (^*^p<0.05).

Our data demonstrated that activated HSCs could suppress T cell responses. However, whether they can inhibit activated T cells remains unknown. To test this, Hepa1-6 cells were labelled using CFSE and used as target cells. As shown in Fig. 5, after gating the CFSE^+^ cells, the proportion of CFSE/PI double-positive cells was lower in the cultures containing HSCs (11.6% ± 2.6%) compared with the no-HSCs group (22% ± 3.2%; 47% decrease; p<0.05). These results demonstrate that activated HSCs markedly reduce the cytotoxic activity of activated T cells.

### The supernatant from MLRs promotes the proliferation and migration of tumour cells

**Figure 6:**
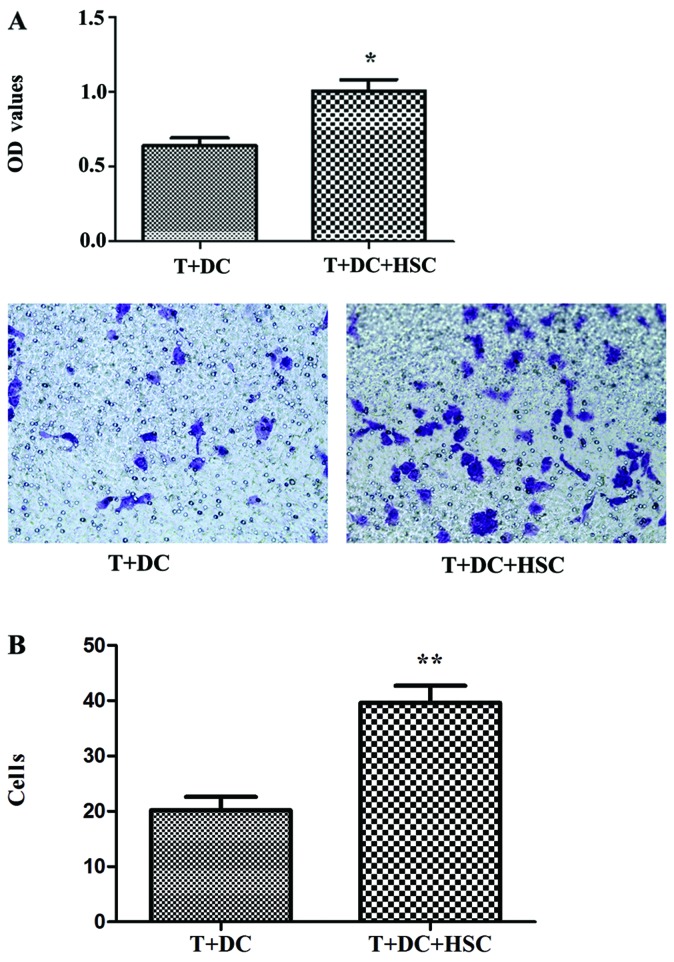
Supernatants from MLRs containing HSC promote HCC cell proliferation and migration. (A) Tumour cell proliferation assay. Hepa1–6 cells were cultured in supernatants from the MLRs for 72 h. Cell proliferation was measured as in the T-cell proliferation experiments. The data are expressed as the mean ± SEM (^*^p<0.05). (B) Tumour cell migration assay. Hepa1–6 cells were added to the upper chamber. Supernatants from the MLRs with or without HSCs were added to the lower chamber. After 18 h, the cells were stained using crystal violet and examined using microscopy (^**^p<0.01).

Since the activated HSCs attenuated the function of T cells, we evaluated the effect of supernatants from MLRs on the proliferation and migration of tumour cells. As shown in Fig. 6A, the supernatant from MLRs containing HSCs clearly promoted the proliferation of Hepa1–6 cells (p<0.05). In addition, the presence of HSCs markedly increased the migration of Hepa1–6 cells (Fig. 6B).

### The expression of suppressive cytokines is altered by HSCs

**Figure 7:**
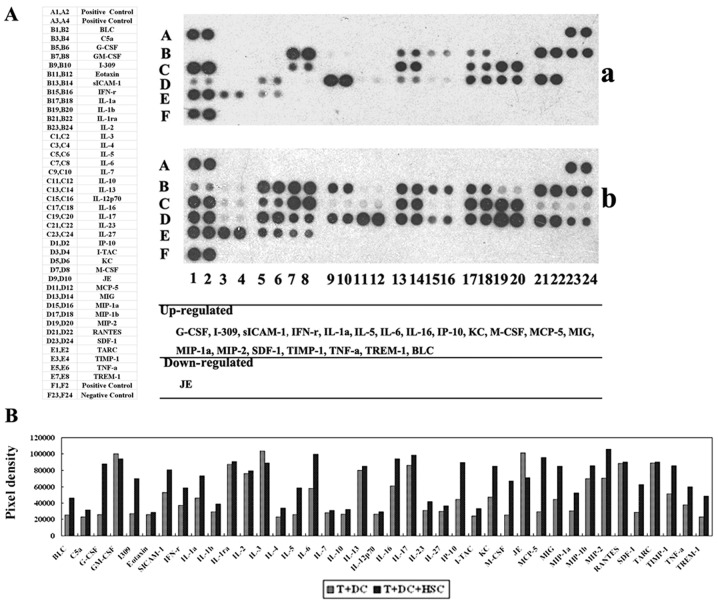
Some immunosuppressive and immunoregulatory cytokines are present in the MLRs supernatants. Supernatants were collected from the MLRs and analysed using a cytokine panel assay. Expression of cytokines with fold change >±1.5 in the T+DC+HSC group are shown. a, T+DC; b, T+DC+HSC. Black dot, positive signal.

HSCs play a role in immunosuppression and immunoregulation not only through their ability to inhibit T-cell responses but also through the secretion of cytokines (19). We evaluated the cytokines in the supernatants from MLRs using a mouse cytokine panel and detected cytokines that induce tolerance in addition to TGF-β. As shown in Fig. 7, the following cytokines were expressed at higher levels in the supernatant from MLRs containing HSCs: B-lymphocyte chemoattractant (BLC), granulocyte colony-stimulating factor (G-CSF), soluble inter-cellular adhesion molecule-1 (sICAM/CD54), interleukin-1α (IL-1α), interleukin-6 (IL-6), chemokine ligand-1 (CCL1/I309), interleukin-16 (IL-16), interferon-γ (IFN-γ), monocyte chemoattractant protein-5 (MCP-5/CCL12), interferon-inducible protein (IP-10/CXCL-10), keratinocyte chemoattractant (KC), monokine induced by IFN-γ (MIG/CXCL-9), macrophage inflammatory protein (MIP-2/CXCL2), MIP-1α, stromal cell-derived factor-1 (SDF-1), tissue inhibitor of metalloproteinase-1 (TIMP-1), tumour necrosis factor α (TNF-α) and triggering receptor expressed on myeloid cells 1 (TREM-1). This suggests that HSCs can also influence the response and cytotoxic capacity of T cells by increasing the expression of some cytokines in the MLRs. These factors may be responsible for the immunosuppressive and immunoregulatory ability of HSCs.

### Activated HSCs promote HCC growth and Treg cells expansion in vivo

**Figure 8:**
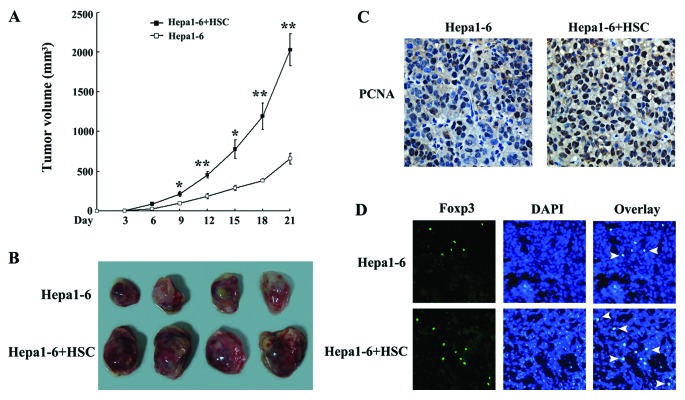
Effect of activated HSCs on HCC cells *in vivo*. (A) Growth kinetics of tumours from C57BL/6 mice after the implantation of Hepa1–6 or Hepa1–6 cells and activated HSCs (^*^p<0.05 and ^**^p<0.01). (B) Representative tumours from C57BL/6 mice 20 days after implantation. (C) Immunohistochemical staining of tumour samples for PCNA. (D) Immunofluorescence staining of tumour sections using FITC-conjugated anti-Foxp3 (green) and DAPI (blue). White arrows indicate representative cells.

To determine the role of HSCs in the development of HCC, we established an *in vivo* model of HCC in mice via the subcutaneous injection of Hepa1–6 cells (control group) or a mixture of Hepa1–6 cells with activated HSCs (experimental group). The tumours grew more rapidly and larger in the experimental group than in the control group (Fig. 8A and B). As HSCs promoted Hepa1–6 proliferation *in vitro* (Fig. 6A), the *in vivo* pro-proliferative response of HSCs was then assessed by analysing tumour samples using PCNA immunostaining. The number of PCNA-positive cells was significantly increased in the experimental group compared with the control group (Fig. 8C). Thus, Treg cells play an important role in tumour immune tolerance. As HSCs increased the expansion of Treg cells *in vitro*, we assessed the effect of HSCs on Treg cells in tumours. As shown in Fig. 8D, the number of Foxp3-positive Treg cells in the experimental group was much higher than the number in the control group.

## Discussion

Following liver injury, HSCs undergo a transformation from quiescent cells containing large retinoid droplets to activated proliferating myofibroblast-like cells. Activated HSCs can promote the development of HCC *in vitro* and *in vivo*(14). However, the mechanisms underlying the impact of HSCs on T cells and the development of HCC remain unclear. We hypothesised that activated HSCs may inhibit the ability of immune cells, especially T cells, to kill cancer cells. Furthermore, HSCs can change the cytokine milieu, which is involved in controlling the immune response to HCC. To gain a better understanding of the underlying mechanism, we utilised one-way MLRs to investigate the effect of HSCs on T cells.

Our findings demonstrated that T-cell proliferation was markedly inhibited in the presence of HSCs compared with control MLRs containing no HSCs and that this effect was dependent on the number of HSCs added. Moreover, we examined the apoptosis of T cells in MLRs via staining with Annexin V and PI and found that most of the T cells were Annexin V/PI double-positive in the MLRs with HSCs. These findings are consistent with other reports (9,11) and demonstrate that activated HSCs suppress the proliferation of T cells and induce the apoptosis of activated T cells. These findings could represent one mechanism through which HSCs modulate the activity of T cells responding to HCC.

A previous study reported that CD4^+^ T cells could be converted to Treg cells through exposure to vitamin A or TGF-β (20). Notably, HSCs store vitamin A and secrete TGF-β in response to inflammation-induced injury. Therefore, it has been suggested that HSCs may exhibit tolerogenic functions in addition to their immunosuppressive ability (21). Our studies confirmed that the number of Treg cells (CD4^+^CD25^+^FoxP3^+^) in the MLRs with HSCs was significantly higher than in the MLRs without HSCs. In addition, the tumours that developed in mice implanted with both HCC cells and HSCs contained more Foxp3-positive cells. These results indicate that HSCs induce an increase in the number of Treg cells, which partially elicit the suppression of the immune response of T cells against cancer cells.

In our study, activated T cells exhibited cytotoxicity against the allogeneic target cells (Hepa1–6 cells) after they were stimulated with DCs exposed to Hepa1–6 lysates. However, this cytotoxicity was blocked by the HSCs. This important finding confirms that activated HSCs attenuate the cytotoxicity of T cells against cancer cells.

The role of activated HSCs in the development of HCC is not only to directly affect T cell function but also to change the expression of cytokines. We analysed the supernatant from the MLRs and found increased expression of a number of cytokines in MLRs containing HSCs, such as IL-6, G-CSF, sICAM-1, SDF-1, IFN-γ and TNF-α. IL-6 is a proinflammatory factor that plays a critical role in the natural history of some malignancies, such as human plasma cell neoplasms, colon cancer, and HCC. IL-6 can mediate autoimmune disease and tumour growth through the IL-6/STAT-3 signalling pathway (22–24).

G-CSF and GM-CSF and their receptors are constitutively expressed in numerous solid tumours, such as skin and head and neck squamous cell carcinomas, gliomas, and meningiomas. Moreover, G-CSF and GM-CSF have previously been shown to stimulate tumour cell growth and migration *in vitro*(25,26).

As a member of the immunoglobulin superfamily, it has been reported that sICAM-1 is immunosuppressive and local release of ICAM-1 appears to promote local immune tolerance and cancer cell immune escape (21,27). ICAM-1 was constitutively expressed on HSCs and can be induced by TNF-α and IFN-γ. ICAM-1 deficient HSCs can partially reverse HSCs immune inhibitory activity both *in vitro* and *in vivo*(9,28). As shown in Fig. 7, higher IFN-γ and TNF-α expression were found in supernatant from MLRs containing the HSCs group. Usually, IFN-γ is a positive regulator in immune reactions. However, after being stimulated by IFN-γ, HSCs become activated, upregulate inhibitory surface molecules B7H1, expand the population of Treg cells, and exhibit profound immunosuppressive activity against the adaptive immune response (11,29).

TNF-α is a multifunctional cytokine involved in apoptosis and cell survival as well as in inflammation and immunity (30,31). Although recognized for its antitumor activity, studies in the pathogenesis of many neurological conditions have demonstrated that TNF-α has immunosuppressive functions during the chronic phase of the disease, suggesting a dual role for TNF-α (32). As a pleiotropic chemokine, SDF-1 has recently been reported to participate in inducing immunological tolerance (33). Although some of the cytokines detected in our study have proinflammatory roles, based on our results, the inhibitory action of these molecules overrides their proinflammatory functions, resulting in T-cell hyporesponsiveness and the metastasis of cancer cells.

In summary, our study demonstrates that activated HSCs can induce the death of activated T cells, modify the type of T cells present (expanding Treg cells), and can reduce the cytotoxicity of cancer-specific T cells. HSCs can also affect the cytokines present in MLRs, resulting in the increased proliferation and migration of cancer cells. Additionally, consistent with the *in vitro* results, activated HSCs can induce HCC proliferation and Treg cell expansion *in vivo*. These results demonstrate that HSCs inhibit the activity of T cells and promote the immune escape of HCC.
